# Supplementary material for: Quantifying the immunological distinctiveness of emerging SARS-CoV-2 variants in the context of prior regional herd exposure
Source: PNAS Nexus. 2022 Jul 4;1(3):pgac105. doi: 10.1093/pnasnexus/pgac105 (PMC9308564; doi:10.1093/pnasnexus/pgac105)
Supplement: pgac105_Supplemental_File [file pgac105_supplemental_file.pdf]

Supplementary Information for:

Quantifying the immunological distinctiveness of emerging SARS-CoV-2 variants in the context of prior regional herd exposure

Michiel J.M. Niesen[1+], Karthik Murugadoss[1+], Patrick J. Lenehan[1+], Aron Marchler-Bauer[2], Jiyao Wang[2], Ryan Connor[2], J. Rodney Brister[2], AJ Venkatakrishnan[1], Venky Soundararajan[1*]

[1] nference, One Main St, East Arcade, Cambridge, Massachusetts 02139, USA

[2] National Center for Biotechnology Information, U.S. National Library of Medicine, National Institutes of Health, Bethesda, MD 20894, USA

+ Joint first authors

*Correspondence to: Venky Soundararajan (venky@nference.net)

Index

Figure S1: Distinctiveness of variants of concern during the time when they first appeared.

Figure S2: Mutational load of variants of concern during the time when they first appeared.

Figure S3. Contribution to Distinctiveness for Spike protein amino acid positions, in India and Brazil.

Figure S4: Correlation between the local Distinctiveness and change in prevalence of a lineage, for all time periods.

Figure S5: Sensitivity analysis for the prediction of future changes in prevalence from average lineage Distinctiveness.

Table S1: Number of sequences per geographic region, included in our analysis.

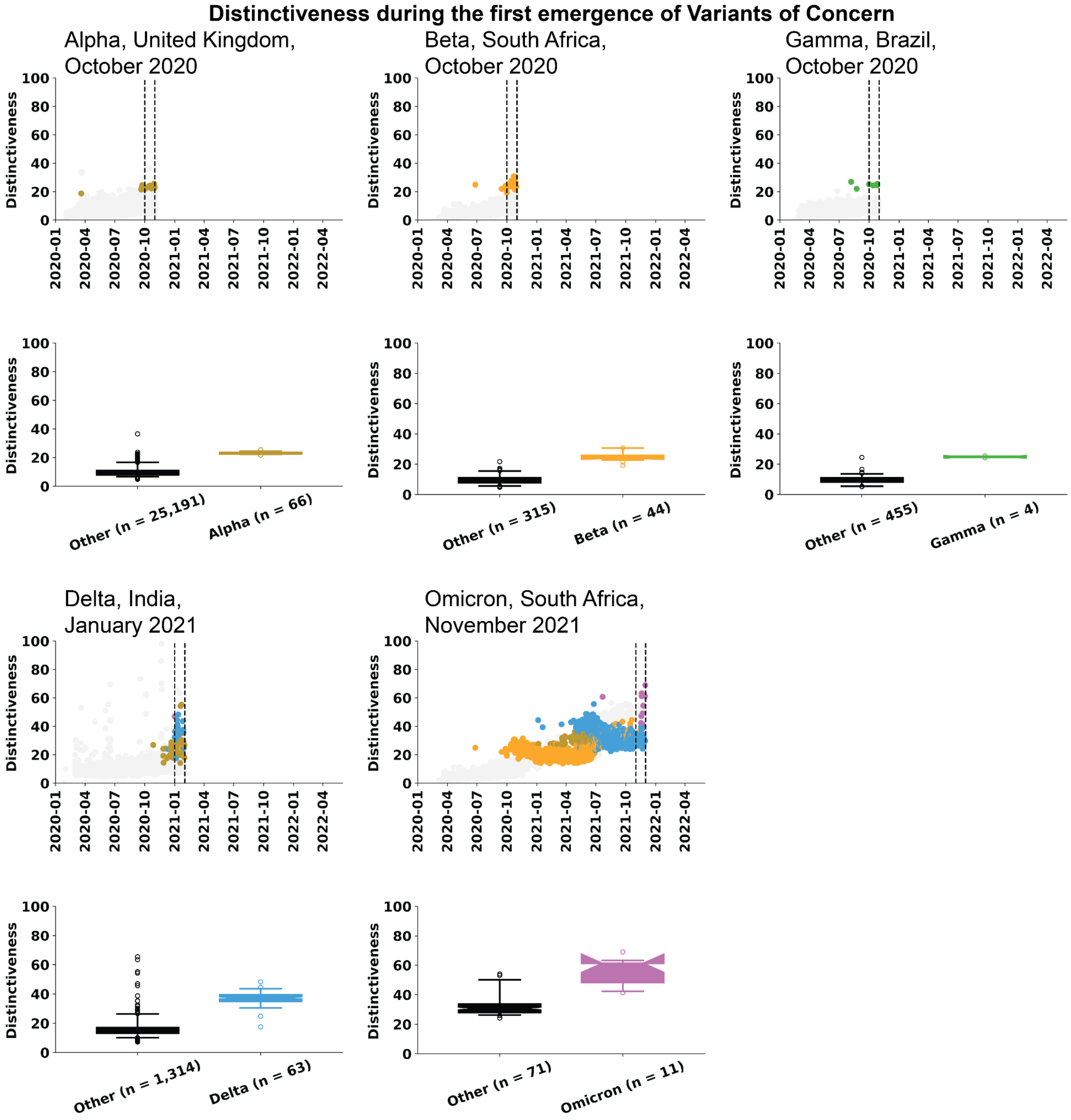

Figure S1: Distinctiveness of variants of concern during the time when they first appeared. In all cases, the Distinctiveness of the VOCs is significantly higher (p-value < 0.001) than that of contemporary sequences. For Alpha, Beta, and Gamma, Distinctiveness values of sequences collected during October 2020 are shown; for Delta Distinctiveness values of sequences collected during January 2021 are shown; and for Omicron Distinctiveness values of sequences collected during November 2021 are shown.

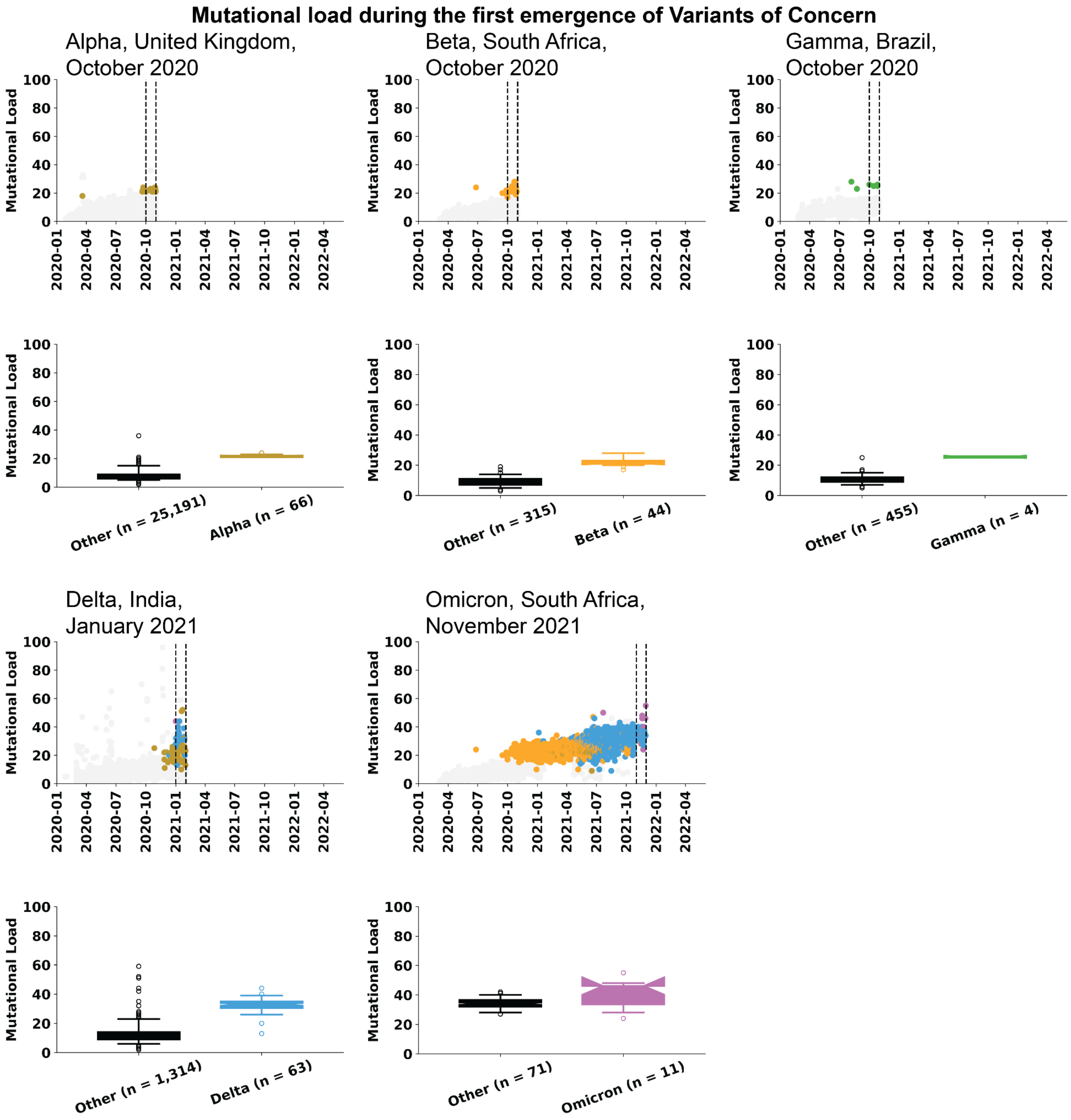

Figure S2: Mutational load of variants of concern during the time when they first appeared. In all cases, the Mutational load of the VOCs is significantly higher (p-value < 0.001) than that of contemporary sequences. For Alpha, Beta, and Gamma, Mutational load values of sequences collected during October 2020 are shown; for Delta Mutational load values of sequences collected during January 2021 are shown; and for Omicron Mutational load values of sequences collected during November 2021 are shown.

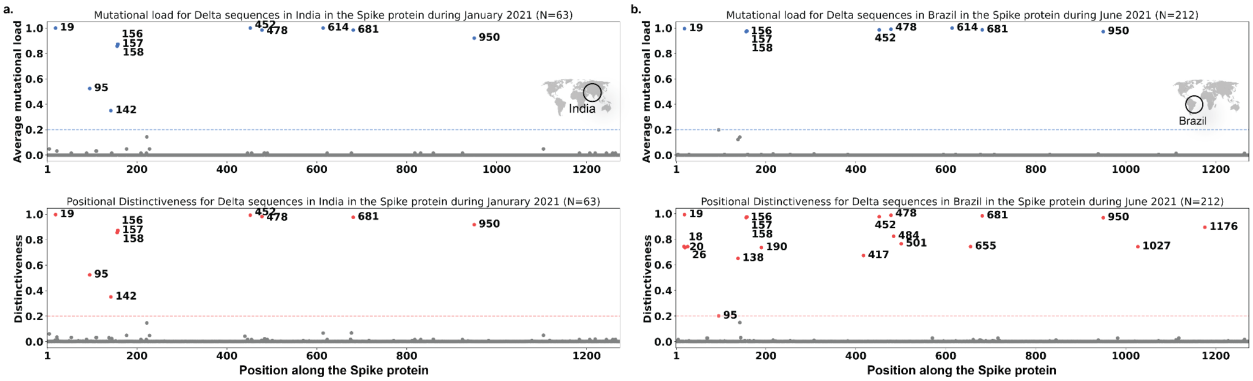

Figure S3. Contribution to Distinctiveness for Spike protein amino acid positions, for Delta sequences in India (**a**) and Brazil (**b**). The x-axes denote the amino acid positions in the Spike protein and the y-axes denote the average mutational load (top panel) or the Distinctiveness (bottom panel). Horizontal lines at y=0.2 denote a high threshold above which amino acid positions are labeled.

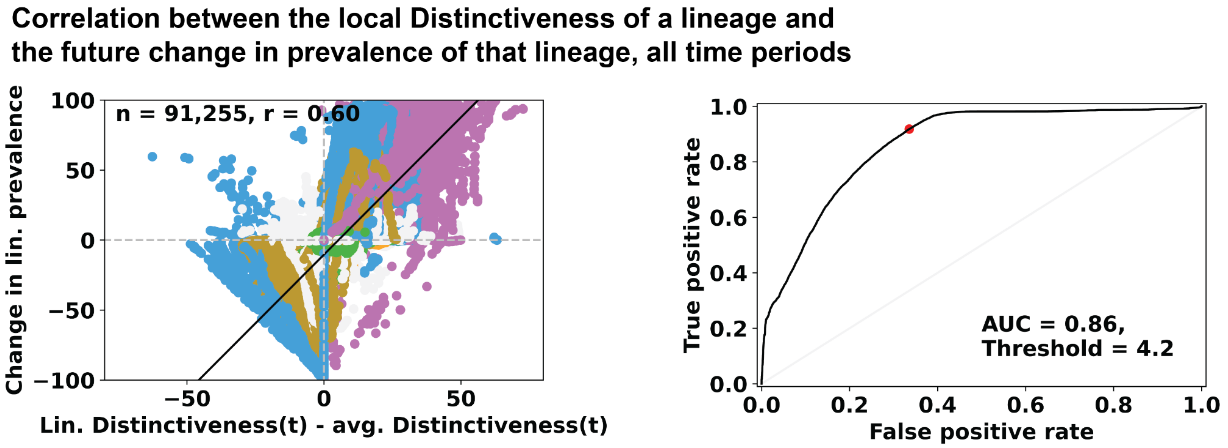

Figure S4: a. Correlation between the change in prevalence of a lineage, from the current time to 56 days in the future, without any filtering of time periods (465 time periods from 78 geographical regions). **b.** ROC for predicting an increase in prevalence of greater than 20 percentage points from an initial 28-day time window and a subsequent 28-day time window, starting 56 days in the future.

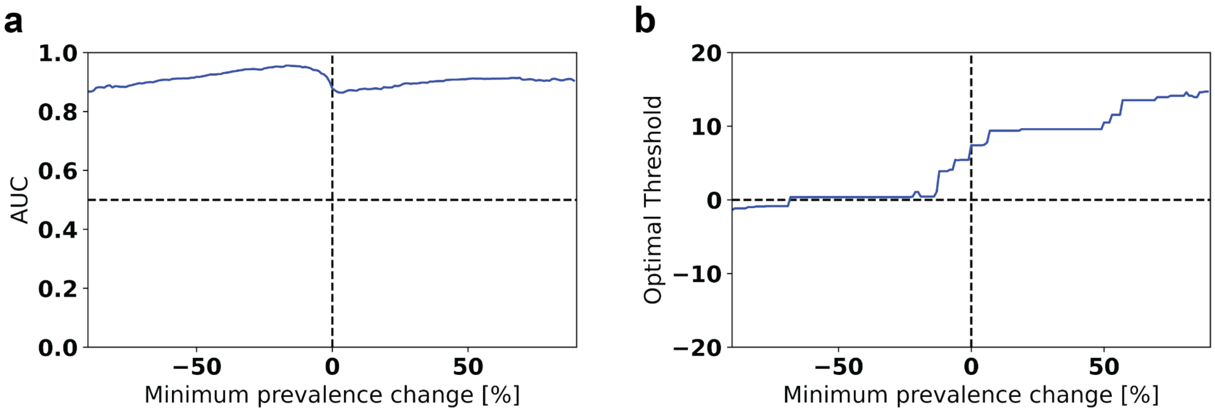

Figure S5: Sensitivity analysis for the prediction of future changes in prevalence from average lineage Distinctiveness. a. AUC for the prediction of lineages with increase in prevalence 56 days in the future from the average Distinctiveness of sequences belonging to that lineage. The minimum increase in prevalence, used to define positive labels, was varied (x-axis), and AUC values were calculated (y-axis). **b.** Optimal threshold values for a positive prediction, using average lineage Distinctiveness relative to contemporary sequences as the predictive variable, that yield the highest sum of Specificity and Sensitivity.

Table S1: Number of sequences per geographic region, included in our analysis.

Country	Sequences from that region
United Kingdom	1,190,215
Denmark	379,340
Germany	290,861
Japan	182,846
Canada	175,571
France	121,274
Sweden	116,432
Brazil	84,477
Switzerland	80,524
Turkey	77,479
Netherlands	76,297
Italy	70,548
Spain	66,644
India	63,720
Belgium	57,909
Norway	45,103
Slovenia	40,782
Australia	36,864
Poland	35,956
Ireland	35,796
Mexico	33,546
Finland	24,028
South Korea	23,891
Lithuania	20,516
Portugal	19,349
Israel	17,106
South Africa	11,362

US state	Sequences from that region
California	266,055
Texas	111,293
New York	88,607
Florida	81,862
Colorado	71,456
Massachusetts	63,438
Minnesota	59,239
Washington	50,713
Illinois	42,502
North Carolina	41,688
Michigan	41,266
Utah	40,007
Pennsylvania	36,626
Arizona	33,575
Wisconsin	32,383
New Jersey	31,301
Georgia	30,652
Maryland	29,086
Ohio	26,979
Oregon	23,820
Tennessee	23,401
Indiana	22,777
Virginia	22,199
Connecticut	18,117
Nevada	17,858
New Mexico	17,512
West Virginia	17,045

US state	Sequences from that region
Louisiana	15,156
Idaho	14,290
Missouri	13,778
Kentucky	13,775
South Carolina	12,508
Kansas	11,199
Alabama	10,947
Iowa	10,727
Wyoming	10,454
Nebraska	10,184
Maine	9,355
Montana	9,069
Rhode Island	8,010
Hawaii	7,889
Vermont	7,493
Arkansas	7,044
Delaware	6,137
New Hampshire	5,942
Mississippi	5,781
Alaska	4,913
North Dakota	3,384
District of Columbia	3,192
Oklahoma	3,025
South Dakota	2,761
